# Seasonal Variations Alter the Impact of Functional Traits on Plankton Dynamics

**DOI:** 10.1371/journal.pone.0051257

**Published:** 2012-12-12

**Authors:** Marcia R. Rocha, David A. Vasseur, Ursula Gaedke

**Affiliations:** 1 Institute of Biochemistry and Biology, University of Potsdam, Potsdam, Germany; 2 Department of Ecology and Evolutionary Biology, Yale University, New Haven, Connecticut, United States of America; 3 Institute of Biochemistry and Biology, University of Potsdam, Potsdam, Germany; Michigan State University, United States of America

## Abstract

Gaining understanding of food-web processes often requires a simplified representation of natural diversity. One such simplification can be based on functional traits, as functionally similar species may provide a similar contribution to ecosystem level-processes. However, understanding how similarity in functional traits actually translates into similar contributions to ecosystem-level properties remains a challenge due to the complex ways in which traits can influence species' dynamics. Moreover, in many communities, seasonality alters the abiotic and biotic forcing regime, causing ongoing changes to patterns of species' dominance; groups of species do not stay intact but are rather continuously subjected to changes throughout the year. Using long-term high frequency measurements of phytoplankton in Lake Constance, we investigated the effect of seasonal changes on the relationship between functional similarity and temporal dynamics similarity of 36 morphotypes, and the relative contribution of different functional traits during the different parts of the year. Our results revealed seasonal differences in the overall degree of synchronization of morphotypes' temporal dynamics and how combinations of functional traits influence the relationship between functional trait similarity and temporal dynamics similarity, showing that different forcing regimes change how species cope with their environment based on their functional traits. Moreover, we show that the individual functional traits matter at different periods of the year indicating that species which are dynamically similar at certain parts of the year may not be at others. The differential strength of the overall and individual impact of functional traits on species' temporal dynamics makes the cohesion of a pair of functionally similar species dependent on the different forcing. Hence, simplifying food webs based solely on functional traits may not provide consistent estimates of functional groups over all seasons.

## Introduction

Investigation into the structure and functioning of natural food-webs often relies upon the aggregation of a high number of species into fewer ecologically meaningful groups [1], [2]. Early work in this area focused on using information from the food web itself, such as the flow of nutrients or energy, to define trophic roles or groups and their similarity and reduce the complexity of ecosystems [3], [4]. However, it was noted that sampling bias within empirical food web data severely limited the use of any singular objective criterion for defining groups [3], [4]. The increasing acknowledgement that species share specific functional properties fostered their description by the biological characteristics required for a species to perform a particular role in the ecosystem through the measurement of functional traits [5], [6], [7], [8], [9]. Suggested functional characteristics range from e.g. basic adaptive strategies such as the C-R-S concept [5] or a performance trait which is the net result of several morpho-physiological traits [6], to specific morpho-physio-phenological traits which directly or indirectly impact fitness [7]. Hence, they constitute potentially a valuable source of information for aggregating species into groups responsible for particular ecosystem-level functions such as N fixation and edible primary production. However, understanding how similarity in functional traits translates into similarity in the contribution to ecosystem-level properties remains a challenge, due to the multitude of potential functional characteristics exhibited by a species and the complex, non-linear ways in which these can interact with a variable environment and other species.

The contribution of a species to ecosystem-level properties is linked to its temporal dynamics, which in turn is determined by the interaction between its functional traits and density-dependent and independent factors. In phytoplankton, the study of the general relationship between functional trait similarity and temporal dynamics similarity has shown that functionally similar species have more similar temporal dynamics than functionally distant ones [9]. However, this general pattern does not necessarily hold all year round in temperate lakes and marine systems, in which plankton communities experience strong seasonal changes in the biotic and abiotic factors which govern dynamics [10]. Seasonality potentially leads to changes in the relative impact of different functional traits on temporal dynamics during different periods. For example, previous work showed that there exists a seasonal alternation between coherent and compensatory dynamics in edible and less edible phytoplankton [11]. During winter when grazing and competition for nutrients are reduced, both groups present coherent dynamics whereas during summer and fall, when both grazing pressure and nutrient limitation are present, edible and less-edible phytoplankton exhibit compensatory dynamics [11]. Moreover, a clear pattern of positive and negative covariances was found within and among functional groups of phytoplankton, mainly driven by different types of predators occurring at different parts of the growing season [12]. This implies that certain functional traits are only important at certain times, suggesting that species which are dynamically similar at certain times are not at others, and challenging the view that functional traits may provide an objective classification for species aggregation in complex food webs.

The structure of phytoplankton assemblages varies greatly according to the seasonal conditions and species may present different strategies based on their tolerances - and thus their functional characteristics - to different combinations of the degree of vertical mixing, light and nutrient availability, competition and grazing [13]. For example, light represents a very important limiting factor for phytoplankton growth during winter, forcing most morphotypes to very low densities. In contrast, competition for nutrients and differential grazing are the dominant factors for phytoplankton growth during summer. To understand how such seasonal changes influence the adequacy of functional traits for defining cohesive groups of functionally similar species throughout the year, we paired long-term high-frequency measurements of phytoplankton in large deep, mesotrophic Lake Constance, with data on the 4 functional traits reflecting the most important growth determining factors in Lake Constance (i.e. maximum growth rate, nutrient demands, susceptibility to grazing and sedimentation) [7]. We determined the relationship between functional trait similarity and temporal dynamics similarity, the relative importance of functional traits for governing dynamics and the overall degree of synchronization among populations for 6 seasonal periods with different abiotic and biotic forcing. Analyzing the impact of seasonality on the relationship between functional trait similarity and temporal dynamics similarity will inform us whether functional similarity is consistently a suitable criterion for simplifying complex food webs throughout the year.

## Methods

### Data Acquisition

**Figure 1:**
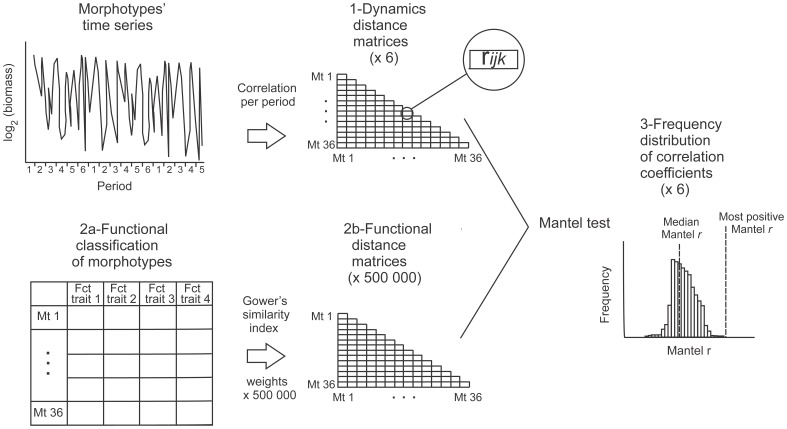
Schematics of the analysis carried out. 1- Dynamical classification of morphotypes (Mt) and quantification of the overall degree of synchronization: for each pair of morphotypes we calculated the average of Pearson correlations per period, the ensemble average 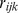, which resulted in 6 dynamics distance matrices, one for each period; 2a- Functional classification of the 36 morphotypes based on 4 functional traits (cell volume, longest linear dimension, silica use and motility) (Weithoff 2003), 2b- Distance matrices based on the traits: 500,000 combinations of weights summing to 1 were attributed to the functional traits. Gower's similarity index was used to calculate the functional distance between the pairs of morphotypes for each set of traits, which resulted in 500,000 functional distance matrices; 3- Comparison of the functional trait similarity and temporal dynamics distance matrices: Mantel test was used to compared the distances matrices culminating in a frequency distribution of the 500,000 different Mantel *r*'s for each period.

Upper Lake Constance (Bodensee) is a large (472 km^2^), deep (depth = 101 m), warm-monomictic temperate lake north of the European Alps. It underwent re-oligotrophication [14] and mean annual phytoplankton biomass declined by a factor of 2 with phosphorous decline, indicating that the long-term changes are small compared to the very pronounced seasonal dynamics (morphotypes vary in density by a factor of 10 to 1000 during the year). Plankton sampling was conducted weekly during the growing season and approximately fortnightly in winter, culminating in 853 sampling dates between 1979 and 1999 (for details see [14], [15]). We log_2_-transformed the biomass measurements to account for the skewed size distribution of phytoplankton organisms [14]. In the present study, we used a taxonomic resolution of 36 morphotypes of phytoplankton comprising individual species or, in some cases, higher taxonomic units which are functionally identical or very similar under the functional classification employed here. This guaranteed a similar taxonomic resolution across the 20 years of sampling, avoided the consideration of a large number of morphotype pairs with (almost) identical traits (functionally “neutral” morphotypes), and reduced a bias towards some groups which were more resolved than others, e.g. because of having particular morphological structures which are lacking in other groups. We treated the non-detection of a morphotype at a particular sampling date as missing value. This approach ensured that we quantified similarity only during periods of temporal co-occurrence rather than during periods when morphotypes were below the detection limit in the plankton or existed only in resting stages. Morphotypes were classified based on their functional traits as well as upon their dynamics, as described below. All steps of our methodology are summarized in Fig. 1.

### Seasonal patterns in Lake Constance

We subdivided the year into 6 consecutive periods (*cf* [16]): winter, early spring, late spring, clear water phase, summer and autumn. The start and end of each period is not a fixed calendar date but was determined for each year based upon independent physical (temperature, vertical mixing, water transparency), chemical (soluble reactive phosphorous concentration) and biological parameters (phytoplankton and zooplankton biomass, chlorophyll concentration and species composition). Other ways of subdividing the annual (e.g. phases fixed by calendar dates) led to phases which were more heterogeneous in respect to their abiotic and biotic conditions throughout the 20 years of sampling and were therefore not reported here. In the approach used here, each period is associated with a different well-defined forcing regime in Lake Constance. (*i*) First, during winter deep mixing and low irradiance lead to a decrease of phytoplankton biomass to the annual minimum level [10]. (*ii*) Early spring follows winter and is characterized by unstable stratification, variable light availability, low grazing pressure and high, non-limiting nutrient concentrations which enables the first growth of algae and small protozoan grazers interrupted by mixing events [17]. (*iii*) During late spring algal biomass further increases with the onset of thermal stratification [10], [18] giving rise to the spring bloom, which reduces nutrient concentrations. Protozoans are the dominant phytoplankton grazers during spring [17]. (*iv*) The high biomass of mostly small, edible algae promotes growth of different groups of micro- and meso-zooplankton [17] and as a consequence, phytoplankton biomass strongly declines, resulting in the clear water phase, which is characterized by the strongest grazing pressure throughout the year [19]. Nutrient concentrations re-increase during the clear water phase and with decreased grazing pressure, the summer phytoplankton bloom starts. (*v*) Summer is marked by severe nutrient depletion leading to strong competition within the phytoplankton community and the presence of different zooplankton groups (ciliates, rotifers, cladocerans and copepods) with different feeding strategies and grazing on different groups of phytoplankton [10], [12], [20], [21]. (*vi*) An increase of the mixing depth as autumn begins leads to a minor reduction of algal biomass and replenishing of nutrients from deeper water. The increase in nutrients may give rise to an autumn phytoplankton and crustacean maximum, paralleled by shifts in algal species composition. Overall, each period is associated with different intensities of abiotic forcing, competition and grazing which are expected to have differentiated effects on the relationship between functional trait similarity and temporal dynamics similarity, the number of important functional traits and their relative importance and the overall degree of synchronization among phytoplankton species.

### Relationship between functional trait similarity and temporal dynamics similarity

#### Functional classification of morphotypes. Selection of functional traits

For phytoplankton, net growth is the sum of intrinsic growth, sedimentation, grazing losses and some other typically less important loss factors. Building on a previous study on functional diversity in Lake Constance, we selected four traits reflecting these three main processes and the 36 morphotypes were classified according to: volume, shape, motility and silica use [7] (nitrogen fixation and mixotrophy were excluded due to a lack of relevance [14], *cf* results).

First, according to allometric theory, size/cell volume strongly influences many physiological attributes such as maximum growth rate [22]. For both colony-forming and single-cell phytoplankton species the classification was done according to individual cell size. Such a classification optimizes the predictability of weight-specific metabolic rates from cell size rather than the vulnerability to grazing and implies that edible and less-edible phytoplankton species strongly overlap in size. Secondly, the shape of a cell or colony (their surface-to-volume ratio) impacts their ability to absorb nutrients, their susceptibility to sedimentation and to filter-feeding zooplankton grazing. In combination with cell volume, a suitable measure for these processes is the longest linear dimension (LLD) of the single cell (for single-cell morphotypes) or the colony (for colony-forming morphotypes). We log_2_-transformed the cell volumes and the LLD values to account for morphotypes skewed size distribution (covering over 4 and 3 orders of magnitude, respectively). Third, motility was considered as mobile organisms can counteract sedimentation and may migrate into favorable strata. In addition, motility affects nutrient deficiency as the movement of cells minimizes the hydrate envelope and, thus, the diffusive boundary layer for nutrients around the cells [23]. Morphotypes were classified as: 0, non-motile; 0.5, buoyancy regulation (through gas vacuoles); and 1, flagellated morphotypes which can move in three-dimensional space [24]. Fourth, silica use was considered as it may be a limiting nutrient in Lake Constance. The use of silica decreases the carbon demand for cell walls and increases the specific weight, leading to higher sedimentation rates. Silica use can be classified as: 1, for diatoms, which need silica for their frustules, 0.5 for Chrysophyceae and Synurophyceae which form statospores (e.g. Ochromonas), bristles and scales (e.g. Synura or Mallomonas) and 0 for all morphotypes which do not use silica [24]. We used the functional traits considered most relevant for our morphotypes [7], however these are obviously a subset of all traits potentially impacting phytoplankton dynamics. We did not consider other commonly used functional traits directly related to light or temperature responses [25]. For light, this is because this information was too unreliable given the plasticity of these functional traits (e.g. taxonomic groups chlorophyll concentration) [25]. Temperature only influences primary production substantially under light saturated conditions [26] [27] which hardly occurs in deep Lake Constance [28].

#### Distance matrices based on the traits

Because our functional traits are of mixed types (continuous and discrete functional traits) we opted for Gower's General Similarity Coefficient, as a single measure of functional distance [29]. To ease interpretation of our results, we used 1-Gower's coefficient as functional metric and defined a pair-wise distance value 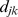 between two morphotypes, as follows:
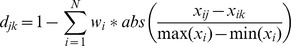
where N is the number of functional traits considered, 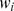 the weight attributed to functional trait i, 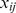 the value of trait i for species j and 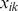 the value of the trait i for species k. Each pair-wise distance ranges from 0 (functionally very different) to 1 (functionally identical). Because we had no *a priori* assumptions about the relative importance of traits, we generated 500,000 random sets of weights summing to 1. This culminated in 500,000 distance matrices based on functional traits differing randomly in their relative importance which were subsequently compared to the 6 distance matrices based on temporal dynamics (Fig. 1).

### Dynamical classification of morphotypes and overall degree of synchronization

For each pair of morphotypes, and within each period, we calculated the Pearson correlation of the time-series of their biomasses. We then averaged these correlations across each period for all years to obtain an ‘ensemble average’ for each morphotype pair in each period (in Fig. 1 referred to as 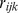 – the ensemble average of the pair of morphotypes i and j, at period k). Ensemble average values range from -1 (perfect compensatory dynamics) to 1 (perfectly synchronized), with 0 representing series which are independent. We excluded periods from the ensemble average in years in which both morphotypes were present together on less than 4 sampling dates. The average number of sampling dates per period was 7.4, 6.2, 6.4, 4.5, 14.2 and 4.8 for winter, early spring, late spring, clear water phase, summer and autumn, respectively. Furthermore we excluded ensemble averages for pairs which yielded a valid correlation (that is, based on at least 4 sampling dates) in less than 20% of all possible periods. These criteria inevitably cause longer periods to present more pairs of morphotypes with valid ensemble averages. For this reason, we also examined the results for only the pairs which have valid correlations for all periods (SI). We constructed 6 distance matrices using the ensemble averages of each period.

The overall degree of synchronization among morphotypes temporal dynamics within a period is quantified by the mean value of the ensemble averages (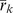, the mean of all ensemble averages 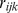, for period k). We tested for differences among these means during different periods using a one-way ANOVA, followed by a Tukey-Kramer post-hoc test for pair-wise comparisons among periods.

### Comparison of the functional and dynamics distance matrices

We compare the set of distance matrices based on the functional traits with the 6 matrices based on dynamics (one for each period) using a Mantel test (package *vegan* under R 2.9.0 [30]). This test determines the Pearson correlation between the two distance matrices (the Mantel *r*, ranging from -1 and 1) and informs us about the nature of the relationship between functional trait similarity and temporal dynamics similarity.

Because we assumed no prior knowledge about the relative importance of traits we examined the distribution of Mantel r's arising in each period from 500,000 randomizations of trait weights (Fig. 1). This allowed us to determine the sensitivity of the relationship between functional distance and dynamics to variation in the importance of different traits. We compared these distributions across the six periods using a Fisher's *z* transform (to normalize our bounded distributions) and then a Wilcoxon rank sum test.

**Figure 2:**
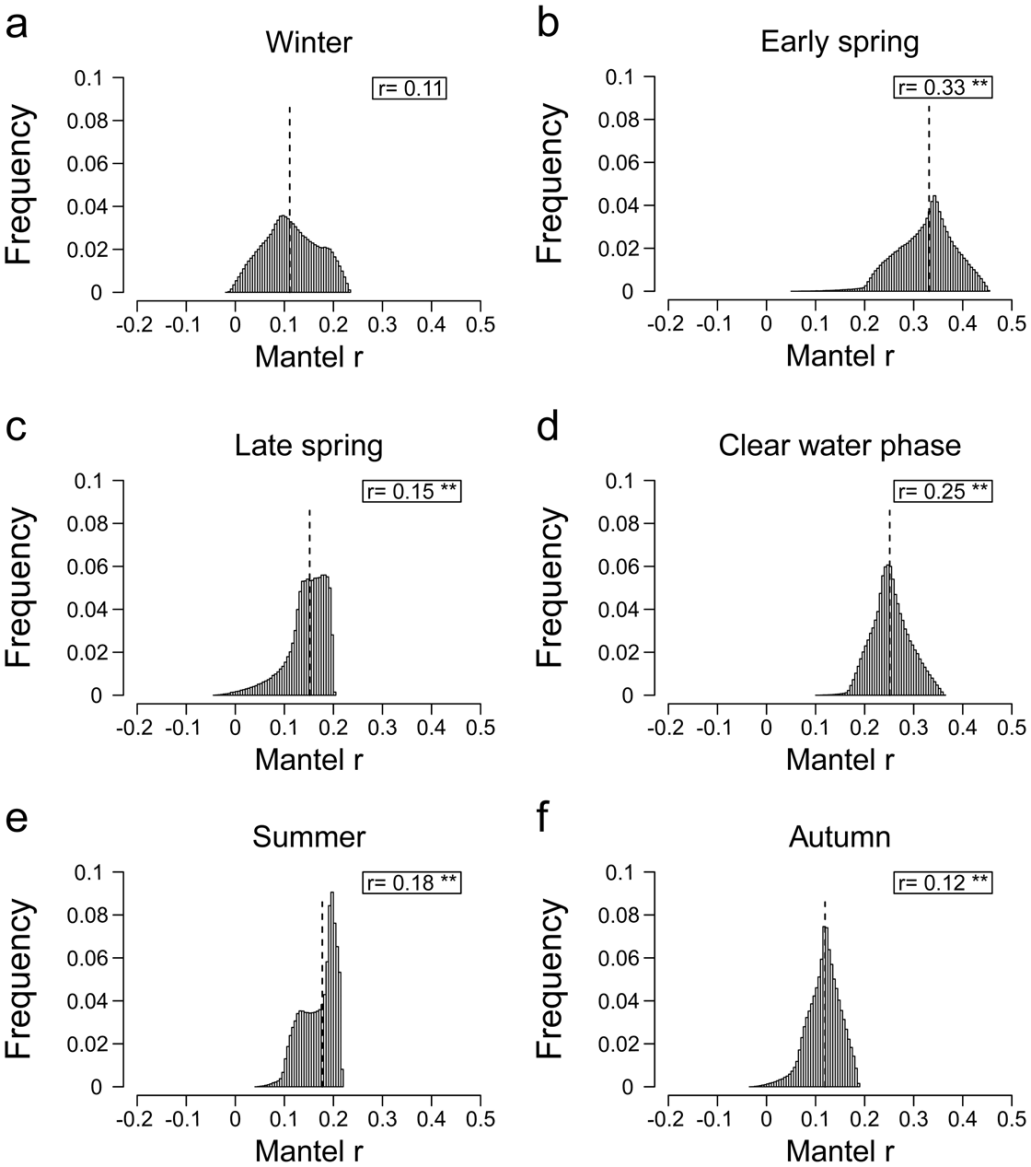
Frequency distribution of 500 000 Mantel *r* per period, based on 500 000 different distance matrices based on functional traits varying in their relative importance. The dashed line indicates the median value of the frequency distribution and the corresponding values are shown in the box within the plot area. ** means p<0.01 and *** means p<0.001. Periods are (a) winter, (b) early spring, (c) late spring, (d) clear water phase, (e) summer and (f) autumn.

**Figure 3:**
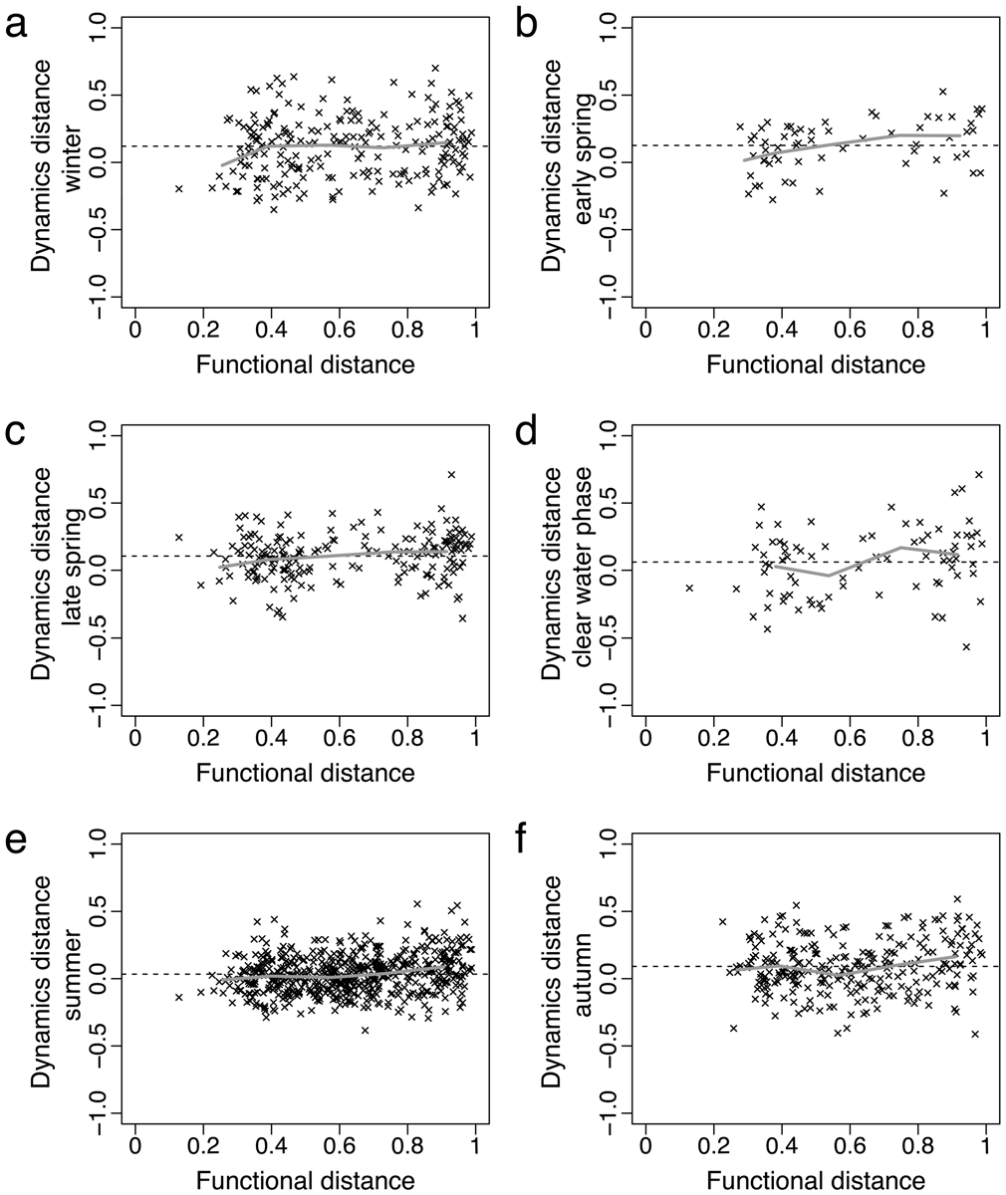
Plots of functional trait distance vs. temporal dynamics distance at different periods of the year. Each dot corresponds to a period ensemble average 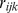, per pair of morphotypes. Functional traits distances are measured for pairs of morphotypes using the Gower distance and temporal dynamic distance is based on a period correlation for (a) winter, (b) early spring, (c) late spring, (d) clear water phase, (e) summer and (f) autumn. The dashed horizontal line indicates the mean of ensemble averages 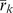 for each period and the solid thick grey trend line represents the running mean.

From each of these six distributions we selected the set of trait weights which gave rise to the median Mantel r as an indicator of the general relationship and used it for further analysis. To determine if the median Mantel r differed significantly from zero in each period, we calculated a null distribution for ‘r’ by randomly shuffling the rows and columns of the morphotypes pairs that occurred together during a given period in the functional distance matrix giving rise to the median Mantel r, and recalculating ‘r’ 10000 times. This procedure is akin to randomly assigning the sets of measured functional traits among species; however, randomizing the functional distances themselves requires fewer computations per replicate. The square of the Mantel *r* quantifies the amount of shared variance between the two distance matrices. It is important to note that we deal with two sorts of correlations throughout this study: first, the correlations between functional similarity and temporal dynamics similarity (the median Mantel *r*, Fig. 2 and Fig. S1) and the temporal correlations among morphotype biomasses (the ensemble averages 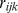, Fig. 3 and Fig. S2).

Moreover, because our data consistently indicated a positive correlation between functional trait similarity and temporal dynamics similarity, we searched for sets of weights resulting in the most positive Mantel *r*. From the 500,000 random sets of weights, we selected all sets which generated the most positive Mantel r (to two decimal places). From these, we assessed the relative importance of each trait, during each period, by calculating the mean and first and third quartiles of the distribution of weights. These sets of weights maximize the functional similarity among morphotype pairs which present more synchronized dynamics and maximize the functional difference among pairs with the least synchronization. In addition, in order to confirm our results, we used an optimization routine to search for the set of weights resulting in the most positive Mantel *r* [31]. We assume that traits which obtain high weights are important for generating dynamic patterns for each one of the periods.

### Variation in pairwise Pearson correlation coefficients per period

**Figure 4:**
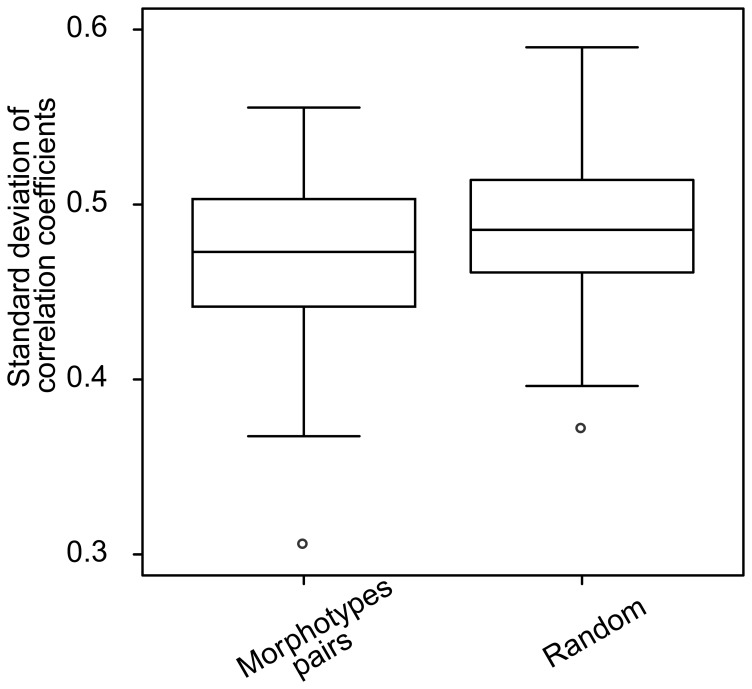
Box plot of the standard deviations of pairwise Pearson correlation coefficients (“Morphotype pairs”) and of the null distribution generated by determining standard deviations from random species pairs and periods (“Random”).

In order to investigate the temporal variation in the Pearson correlation coefficients giving rise to the period ensemble averages 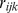, we studied their standard deviations (generated from across-year replicates). The goal of this analysis was to test whether species pairs typically display consistent correlation coefficients throughout the study period or whether the extent of correlation within one species pair is as variable as expected from random pairings. First, we calculated the standard deviation of the Pearson correlation coefficients (for all periods across years) for the 119 pairs of morphotypes which occurred together in at least 25% of the sampling dates, culminating in 119 standard deviations (each of those calculated from *n* Pearson correlation coefficients). We compared these to a null distribution where we randomly selected *n* Pearson correlations coefficients from all 119 morphotype pairs and all periods (Fig. 4). We applied Fisher *z* transformation to all coefficients. We used a t-test to compare the observed and null distributions. All metrics were coded using R 2.9.0 [30].

No specific permits were required for the described field studies. Herewith we confirm that the location is not privately-owned or protected in any way. We also confirm that the field studies did not involve endangered or protected species.

## Results

Functionally similar morphotypes presented more similar dynamics than functionally different morphotypes in all periods except winter. Furthermore, the frequency distributions of Mantel *r*'s per period significantly differed from each other (p<0.001, Wilcoxon rank-sum test) (Fig. 2), demonstrating that functional similarity has the capacity to explain different amounts of the variation in dynamics similarity in different periods. In each period, the median values of these distributions were significantly greater than zero (in all but winter; p<0.01) (Fig. 2) indicating that an increase in functional similarity is accompanied by a strengthening in the covariation of morphotypes temporal dynamics. The median Mantel *r*'s amounted to 0.11, 0.33, 0.15, 0.25, 0.18 and 0.12, for winter, early spring, late spring, clear water phase, summer and autumn, respectively. The amount of shared variance between distance matrices measuring similarity in functional traits and distance matrices measuring similarity in temporal dynamics, which can be quantified by the square of the Mantel r's, varied between 1.5 and 10% (Fig. 2 a–f).

Since the seasonal periods have different lengths and sampling frequencies, periods differ in their number of valid ensemble averages 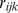. In addition, the sets of pairs of morphotypes also change among periods. This may lead to differences between periods based only on the number of morphotypes and/or morphotype composition. To control for this we repeated the computations with matrices comprising the same morphotype pairs (i.e. those which were present during all periods). There were less than 50 pairs out of 630 in common among all 6 periods and the morphotypes present in winter and early spring were particularly different from those present in the remaining periods. Late spring, clear water phase, summer and autumn presented 62 pairs of morphotypes in common, and we hence performed this analysis using only these 4 periods. Overall, consistently high values of median Mantel *r* (0.41, 0.3, 0.42 and 0.4 during late spring, clear water phase, summer and autumn, respectively) (Fig. S1) point to a relatively stronger correlation between functional trait similarity and temporal dynamics similarity quantified when considering only the common pairs of morphotypes. Moreover, the frequency distributions of Mantel *r* were significantly different from each other (p<0.001, Wilcoxon rank-sum test) (Fig. S1).

Regarding the overall degree of synchronization of morphotypes dynamics per period, we found a significant difference between the means of the ensemble averages per period 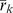 (F = 12.3, p<0.001) (Table 1). The overall degree of synchronization 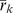 during winter, early spring, late spring and autumn was significantly higher than during summer (p<0.05) and the clear water phase presented a significantly lower overall degree of synchronization among pairs of morphotypes than winter (p<0.05) (Fig. 3 and Table 1 - Tukey-Kramer post hoc test). The clear water phase showed the most negative ensemble averages 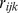 among all periods and summer presents the highest number of ensemble averages 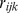 close to 0 (Fig. 3). When considering only the common pairs of morphotypes between late spring and autumn, the means of the ensemble averages per period 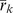 did not significantly differ (F = 2.1, p = 0.15) (Fig. S2).

**Table 1:** Mean period ensemble average (F = 12.26, p<0.0001).

	Winter	Early spring	Late spring	CWP	Summer	Autumn
Mean period ensemble average	0.12^a^	0.13^a,b^	0.1^a,b^	0.06^b,c^	0.03^c^	0.09^a,b^

Different superscript letters indicate significant difference among periods using a Tukey Kramer post hoc test. CWP: Clear water phase.

For both the analysis including all data and the one including only the common pairs of morphotypes, the relationship between functional trait similarity and temporal dynamics similarity was not associated with a specific set of weights for the different traits, but rather very different sets of traits led to the same median Mantel *r*. In contrast, although the trait longest linear dimension had consistently the largest weight, we found that the set of weights leading to the most positive Mantel *r* was unique per period but variable among periods, showing that different functional traits matter at different times of the year (Table 2 and Table S1). The combination of weights generating the most positive Mantel *r* was little affected by either using all data or considering only the pairs of morphotypes present from late spring to autumn except for late spring, during which the relative importance of traits changed strongly under the more restrictive rules). Moreover, different ways of subdividing the year into phases led to comparable results but as they typically comprised more heterogeneous abiotic and biotic conditions difference between periods in Mantel *r* and in the relative importance of trait weights were typically smaller. Both the maximization of Mantel *r* and the study of the distributions of Mantel *r* with varying weights arrived at the same answer concerning the weights leading to the most positive Mantel *r*.

**Table 2:** Mean and standard deviation of weights leading to the most positive Mantel *r* per period.

	Median Mantel r	Traits				Most positive Mantel r
		Cell volume	LLD	Silica use	Motility	
Winter	0.11	0.43 (0.34; 0.52)	0.45 (0.37; 0.54)	0.10 (0.06; 0.13)	0.02 (0.01; 0.03)	0.19**
Early spring	0.33**	0.04 (0.01; 0.05)	0.73 (0.69; 0.76)	0.20 (0.16; 0.23)	0.03 (0.02; 0.05)	0.42***
Late spring	0.15**	0.03 (0.01; 0.04)	0.29 (0.21; 0.38)	0.65 (0.55; 0.73)	0.03 (0.01; 0.05)	0.20**
CWP	0.25**	0.05 (0.02; 0.08)	0.73 (0.69; 0.76)	0.03 (0.01; 0.04)	0.19 (0.16; 0.22)	0.35***
Summer	0.18**	0.15 (0.07; 0.22)	0.38 (0.31; 0.46)	0.36 (0.31; 0.42)	0.10 (0.06; 0.14)	0.22***
Autumn	0.12**	0.05 (0.02; 0.08)	0.63 (0.58; 0.68)	0.25 (0.2; 0.3)	0.06 (0.03; 0.09)	0.21***

** means p<0.01.

*** means p<0.001.

LLD: Longest linear dimension. CWP: Clear water phase.

The standard deviations of the Pearson correlation coefficients of the individual morphotype pairs were significantly less than those drawn at random from different morphotype pairs (p<0.001, n = 119). However, the means only differed by very little indicating a large temporal variability in the dynamics patterns of numerous pairs of morphotypes (Fig. 4).

## Discussion

The study of morphotype dynamics under different regimes of biotic and abiotic forcing reveals differences in how functional traits influence dynamics and in the relative importance of traits within each period. The distributions of correlations among functional similarity and dynamic similarity, generated by randomly weighting the importance of functional traits, are significantly different across periods. Because these distributions do exhibit some degree of overlap, we cannot rule out the possibility that the community might adhere to a set of trait weighting which produces similar correlations across some or all periods. However, explicitly addressing this issue would require an *a priori* knowledge of the ‘correct’ weighting scheme during each period, which is not available. Moreover, it is likely that the relative importance of traits changes continuously through time and varies from year to year based on biotic and abiotic conditions. Our results demonstrate the capacity for differences among periods without such an *a priori* knowledge of the importance of traits and we suggest that these differences are driven by the biotic and abiotic factors impacting dynamics. Overall, morphotypes with high functional trait similarity present dynamics which are more similar than those of dissimilar morphotypes, as shown by the positive median Mantel *r* encountered for all periods except winter for which median Mantel *r* is not significantly different from zero.

Winter is characterized by a non-significant relationship between functional trait similarity and temporal dynamics similarity indicating that the traits used here have little influence on morphotypes' dynamics. The high overall degree of synchronization found during this period (given by the 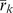) suggests that the strong external forcing, i.e. low light and deep mixing, affect all morphotypes in concert regardless of their functional traits and lead them to decrease in abundance. This may reduce the predictive power of the functional traits considered here for morphotypes' temporal dynamics.

During early spring, we found a positive relationship between functional trait similarity and temporal dynamics similarity and the highest degree of synchronization, showing that even though changes in the temporary stratification due to strong mixing events tend to synchronize morphotypes dynamics, functional traits also play an important role in determining the dynamics. During late spring, the relationship between functional trait similarity and temporal dynamics similarity was also significantly different from zero, indicating that functional traits influence morphotypes' dynamics.

As a result of intensified grazing pressure after late spring, phytoplankton biomass declines rapidly which gives rise to the clear water phase, during which only fast-growing (typically small) species persist. This period is characterized by a positive median Mantel *r*, indicating that functional traits influence dynamics during this period. In contrast to the earlier periods of the year, the overall degree of synchronization is low during the clear water phase, which is due to strong negative correlations between several pairs of morphotypes during this period: whilst edible plankton decline due to grazing, some less edible morphotypes start growing benefitting from increased light and nutrient availability.

During summer, functional traits play a strong role as indicated by a highly significant positive median Mantel *r*. In addition, the temporal dynamics of numerous pairs of morphotypes are uncorrelated leading to the lowest overall degree of synchronization throughout the year when considering all pairs of morphotypes. These findings may be a result of e.g. different zooplankton groups exerting their grazing pressure on different phytoplankton groups [12] and the different strategies displayed by species to cope with nutrient depletion.

An increase of the mixing depth as autumn begins leads to replenishment of nutrients from deeper water and a deterioration of the underwater light climate. During this time, along with a positive significant median Mantel r, we found an increase in the overall degree of synchronization, likely due to events of deep mixing, nutrient pulses and insufficient light which may affect morphotypes in concert.

The sets of trait weights leading to the most positive Mantel *r* changed throughout the year. Longest linear dimension was important all year round, being most of the time (except late spring) the single most important functional trait we included. Cell volume was important only during winter and summer but not during the other periods. The lower relevance of cell size may be explained by the fact that our classification of cell size according to individual cell size and not to colony size implies that edible and less-edible phytoplankton species strongly overlap in size. In contrast, the longest linear dimension of the cell or colony/filament is more strongly related to the susceptibility to zooplankton grazing. This underlines the importance of grazing for determining morphotypes' temporal dynamics in Lake Constance as found by e.g. [12]. Silica use reaches its highest weight during late spring, when silica-demanding morphotypes dominate. Interestingly, we found that in summer all functional traits matter in contrast to the other periods. Different groups of zooplankton with different food selectivity graze on phytoplankton during summer [12], [19], [21], synchronizing the dynamics of species with similar cell volume and longest linear dimension. Moreover, summer is characterized by the strongest nutrient scarcity throughout the year and a sequential depletion of nutrients shifts algal composition, namely diatoms dynamics may be regulated by the changes in silica concentrations, which may explain the high importance of the trait “silica use” during summer. Motility matters as it is directly or indirectly associated with several factors impacting the dynamics, notably nutrient deficiency and sedimentation which are most relevant during summer. Besides summer, motility also matters during the clear water phase.

The prevalence of positively correlated temporal dynamics among pairs of morphotypes is in agreement with previous studies which showed that synchronized dynamics dominate in natural systems [32]. However, the majority of positive ensemble averages encountered in our study are rather weak (Fig. 3) and we mostly found no consistent pattern of covariation for a pair of morphotypes throughout the year and a large interannual variability (Fig. S3). Negative ensemble averages are also frequent between pairs of morphotypes, indicating the presence of compensatory dynamics. Compensatory dynamics have been previously quantified in the Lake Constance phytoplankton and attributed to the interaction between edible and less-edible groups, which is mediated by competition for nutrients and grazing during the growing season [11], [12]. The negative correlations spread along the entire axis of functional distance which indicates that these processes occur equally among functionally similar and different morphotypes. To the extent that negative correlations reflect competitive interactions, this can be explained by the fact that all phytoplankton morphotypes compete for the same essential resources (e.g. light and phosphorous required for photosynthesis and growth) regardless of their functional traits [33] and that edible and less edible forms can differ substantially in other traits. Here we vary the functional distance of a pair of morphotypes by applying different sets of weights to functional traits and find that different traits matter at different times of the year, because the dynamical patterns of a pair of morphotypes are also variable across seasons. Moreover, the pairwise correlations (giving rise to the ensemble average 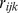) within one pair of morphotypes often alternate between synchronized and compensatory dynamics (Fig. S3), showing that the degree of functional similarity of a morphotype pair does not determine alone how the pair will covary in time. The rather weak but significant relationship between functional trait similarity and temporal dynamics similarity we encountered is, however, reassuring of the effect traits have on dynamics. The interplay of all these factors including functional similarity and their impact on the covariation patterns of pairs of species under different forcing regimes represents a promising area for future research.

Previous work showed that functional redundancy plays a key role in measures of functional diversity [34]. We show that different functional traits are important for determining morphotypes' temporal dynamics in different parts of the year, suggesting that morphotypes which are redundant during one part of the year may not be redundant at others. This annual reorganization in the extent of functional differences among morphotypes may lead to considerable changes in functional diversity. Our results suggest that the common approach (but see also [35]) for calculating functional diversity (considering functional traits with fixed relative importance) will likely profit from a more detailed consideration of the distinct periods of the year, which not only vary in species composition and richness but also in the relative importance of traits and in the extent of functional redundancy.

Our seasonally resolved study suggests that functional similarity is a valid criterion to group species based on e.g. their trophic roles [3], [4] into aggregate entities, since functionally similar species generally have more similar temporal dynamics than functionally distant species ([9] and present results). However, we also show that the link between functional traits and temporal dynamics changes throughout the year, indicating that species aggregations may periodically reorganize. Moreover, the range of functional characteristics we assess here is small compared to the potential range contained in an entire food web. Thus, when larger functional distances are taken into account, the cohesion among species within a group may become even weaker unless the additional functional characteristics have greater importance than those we have considered here. We conclude that functional similarity is a useful predictor of dynamic similarity and thereby can be useful for assigning species to aggregate groups. However, given the seasonal variability in the importance of functional traits functional similarity should not be used as the sole means to group species into higher aggregation levels. More research of this kind, both theoretical and empirical, is needed to further evaluate the criteria for species aggregations.

## Supporting Information

Table S1 Mean and standard deviation of weights leading to the most positive Mantel r per period, for the pairs of morphotypes in common between late spring, clear water phase, summer and autumn. ** means p<0.01 and *** means p<0.001. LLD: Longest linear dimension and CWP: clear water phase.
(DOC)

Figure S1 **Frequency distribution of 500 000 Mantel** ***r*** **per period, based on 500 000 different distance matrices based on functional traits varying in their relative importance, using only the pairs of morphotypes consistently present between late spring and autumn (n = 62).** The dashed line indicates the median value of the frequency distribution and the corresponding values are shown in the box within the plot area. *** means p<0.001. Periods are (a) late spring, (b) clear water phase, (c) summer and (d) autumn.
(TIF)

Figure S2
**Plots of functional trait distance vs. temporal dynamics distance at different periods of the year, only for the pairs of morphotypes consistently present between late spring and autumn (n = 62).** Each cross corresponds to a period ensemble average 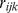, per pair of morphotypes. Functional traits distances are measured for pairs of morphotypes using the Gower distance and temporal dynamics distance is based on a period correlation for (a) late spring, (b) clear water phase, (c) summer and (d) autumn. The dashed horizontal line indicates the mean of ensemble averages 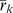 for each period (0.14, 0.08, 0.08 and 0.13 for late spring, clear water phase, summer and autumn, respectively) and the solid thick grey trend line represents the running mean.(TIF)

Figure S3Correlation coefficients per period, for three pairs of morphotypes: a. *Chrysochromulina parva* and *Erkenia subaequiciliata* (both motile algae with similar cell size and longest linear dimension, showing high functional similarity); b. *Asterionella formosa* and *Erkenia subaequiciliata* (a colony forming diatom and a motile chrysophyte, respectively, with low functional similarity) and c. *Rhodomonas* spp and *Cryptomonas* spp (both motile algae with rather similar cell volume and longest linear dimension (high functional similarity) which were present together in 97% of all sampling dates). These correlation coefficients give rise to the pairwise period ensemble averages 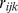, represented here by the tickmarks on the right axis (the color corresponds to the colors of the periods). We observe that correlation coefficients are highly variable within a pair of morphotypes in the different periods of the year. However, functional similarity leads to differences in the period ensemble averages. The functionally more similar pairs (a. *Chrysochromulina parva* and *Erkenia subaequiciliata* and c. *Rhodomonas* spp and *Cryptomonas* spp) are mainly positively correlated throughout the year, whereas the functionally most different pair (b. *Asterionella formosa* and *Erkenia subaequiciliata*) presents a wide range of positive and negative correlations over all periods, leading to ensemble averages closer to 0. Moreover, we find that correlation coefficients vary in different ways during the different periods. Note that two tickmarks are missing (early spring in a. and clear water phase (CWP) in b., as correlations were valid in less than 20% of all possible periods).(TIF)
