# Supplementary material for: Seasonal Variations Alter the Impact of Functional Traits on Plankton Dynamics
Source: PLoS One. 2012 Dec 12;7(12):e51257. doi: 10.1371/journal.pone.0051257 (PMC3523777; doi:10.1371/journal.pone.0051257)
Supplement: Table S1 — Mean and standard deviation of weights leading to the most positive Mantel r per period, for the pairs of morphotypes in common between late spring, clear water phase, summer and autumn. ** means p<0.01 and *** means p<0.001. LLD: Longest linear dimension and CWP: clear water phase. (DOC) [file pone.0051257.s001.doc]

**Supporting Information**

|  | Median Mantel r | Traits | | | | Most  positive Mantel r |
| --- | --- | --- | --- | --- | --- | --- |
|  |  | Cell volume | LLD | Silica use | Motility |  |
| Late spring | 0.43** | 0.03 (0.01; 0.05) | 0.64 (0.6; 0.67) | 0.22 (0.18; 0.25) | 0.11 (0.08; 0.14) | 0.48*** |
| CWP | 0.31** | 0.05 (0.02; 0.07) | 0.75 (0.72; 0.78) | 0.02 (0.01; 0.03) | 0.18 (0.16; 0.2) | 0.42*** |
| Summer | 0.44** | 0.21 (0.17; 0.26) | 0.48 (0.43; 0.53) | 0.25 (0.22; 0.27) | 0.06 (0.04; 0.06) | 0.48*** |
| Autumn | 0.42** | 0.02 (0.01; 0.02) | 0.52 (0.47; 0.56) | 0.22 (0.18; 0.27) | 0.24 (0.2; 0.28) | 0.47*** |
